# Developmental and oncogenic effects of Insulin-like Growth Factor-I in Ptc1^+/- ^mouse cerebellum

**DOI:** 10.1186/1476-4598-9-53

**Published:** 2010-03-09

**Authors:** Mirella Tanori, Melissa Santone, Mariateresa Mancuso, Emanuela Pasquali, Simona Leonardi, Vincenzo Di Majo, Simonetta Rebessi, Anna Saran, Simonetta Pazzaglia

**Affiliations:** 1 Section of Toxicology and Biomedical Sciences, ENEA CR-Casaccia, Rome, Italy

## Abstract

**Background:**

Medulloblastoma is amongst the most common malignant brain tumors in childhood, arising from neoplastic transformation of granule neuron precursors (GNPs) of the cerebellum *via *deregulation of pathways involved in cerebellar development. Deregulation of the Sonic hedgehog/Patched1 (Shh/Ptc1) signaling pathway predisposes humans and mice to medulloblastoma. In the brain, insulin-like growth factor (IGF-I) plays a critical role during development as a neurotrophic and neuroprotective factor, and in tumorigenesis, as IGF-I receptor is often activated in medulloblastomas.

**Results:**

To investigate the mechanisms of genetic interactions between Shh and IGF signaling in the cerebellum, we crossed nestin/IGF-I transgenic (IGF-I Tg) mice, in which transgene expression occurs in neuron precursors, with *Ptc1*^*+/- *^knockout mice, a model of medulloblastoma in which cancer develops in a multistage process. The IGF-I transgene produced a marked brain overgrowth, and significantly accelerated tumor development, increasing the frequency of pre-neoplastic lesions as well as full medulloblastomas in *Ptc1*^*+/-*^/IGF-I Tg mice. Mechanistically, tumor promotion by IGF-I mainly affected preneoplastic stages through *de novo *formation of lesions, while not influencing progression rate to full tumors. We also identified a marked increase in survival and proliferation, and a strong suppression of differentiation in neural precursors.

**Conclusions:**

As a whole, our findings indicate that IGF-I overexpression in neural precursors leads to brain overgrowth and fosters external granular layer (EGL) proliferative lesions through a mechanism favoring proliferation over terminal differentiation, acting as a landscape for tumor growth. Understanding the molecular events responsible for cerebellum development and their alterations in tumorigenesis is critical for the identification of potential therapeutic targets.

## Background

Normal development and tumorigenesis have several common characteristics. In particular, pediatric neoplasms of the nervous system, arising from progenitor cells which are already proliferating as part of the developmental process, are closely linked to disordered mechanisms of normal development. The delicate balance among programmed cell death, proliferation and differentiation, in fact, is crucial for normal neural development. Defects in any of the mechanisms controlling these processes could promote transformation, making developing cells prone to tumorigenesis.

Medulloblastoma is the most common pediatric brain tumor, and develops in the cerebellum of children and young adults. Expression profiling of medulloblastoma has indicated a remarkable similarity between this tumor and early postnatal cerebellum, arguing that the germinal layer of the cerebellum harbors precursor cells for medulloblastoma [1,2]. During cerebellar development, granule neuron precursors (GNPs) migrate from the rhombic lip to the external granular layer (EGL), where they postnatally undergo a proliferative burst before exiting the cell cycle and migrating inward to form the mature inner granule layer (IGL). The cerebellum undergoes an over 1000-fold increase in volume during this process [3]. Proliferation of GNPs is governed principally by the Sonic hedgehod pathway (Shh), but their expansion and survival are also promoted by insulin-like growth factors (IGFs).

Deregulation of the Shh pathway has been linked to medulloblastoma development. Approximately 15-30% of sporadic medulloblastomas contain mutations in *Patched1 *(*Ptc1*) or other elements of the Shh pathway [1,4,5]. Germline deficiency of the Shh receptor, Ptc1, is responsible of the hereditary Nevoid Basal Cell Carcinoma Syndrome (NBCCS) in which patients are predisposed to medulloblastoma and other tumors. Mice with heterozygous *Ptc1 *mutations are also susceptible to medulloblastoma formation, and 8-40% of them develop tumors that resemble human medulloblastomas [6,7]. These mice have provided information on the early stages of tumorigenesis [8,9] and on the genes that cooperate with deregulation of the Shh pathway to promote tumor progression [10-12].

IGF-I and IGF-II act as potent survival factors expressed in a wide variety of cell types. IGF signaling is important for central nervous system (CNS) development, and increased IGF-I activity results in brain overgrowth [13,14]. Moreover, molecular oncology studies in humans and mice strongly implicate IGFs in medulloblastoma development.

In this study, to clarify the role of IGF-I in physiological (development) and pathological (tumorigenesis) settings in the cerebellum, we cross-bred transgenic mice, overexpressing IGF-I (IGF-I Tg) in neural progenitors under control of regulatory sequences from the *nestin *gene [15], with *Ptc1*^*+/- *^mice, a faithful model of human medulloblastoma.

## Results

### Expression of IGF-I transgene and nestin in the cerebellum

**Figure 1:**
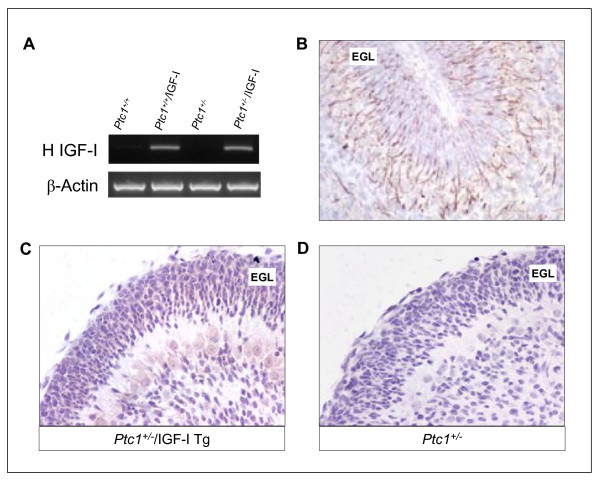
**Analysis of nestin/IGF-I transgene expression in mouse cerebellum at P5**. (A) Quantification of expression of human (H) IGF-I transgene by reverse-transcription PCR with relative β-actin to control cDNA loading. (B) Immunostaining for nestin, showing a marked expression in the EGL, and in other layers of the developing cerebellum. (C) Immunostaining for human IGF-I, showing expression in the cerebellum of *Ptc1*^*+/-*^/IGF-I Tg mice and lack of expression in the cerebellum of *Ptc1*^*+/- *^mice (D).

To examine the impact of IGF-I overexpression on normal development and tumorigenesis in the cerebellum, we crossed *Ptc1*^*+/- *^mice with IGF-I Tg mice [15]. Expression of the human IGF-I transgene, quantified by reverse-transcription PCR in cerebella at P5, was evident in the cerebellum of *Ptc1*^*+/+*^/IGF-I Tg and *Ptc1*^*+/-*^/IGF-I Tg mice, whereas it was absent in *Ptc1*^+/+ ^and *Ptc1*^*+/- *^cerebella (Figure 1A). Since the spatial expression pattern of nestin/IGF-I transgene was reported to be consistent with that of the *nestin *native gene [16], to localize the expression of the transgene, sections of cerebellum from mice at P5 were immunostained for nestin. As shown in Figure 1B, nestin was strongly expressed in GNPs of the EGL, as well as in other layers of the developing cerebellum. In addition, by immunostaining, with an antibody that specifically recognizes human IGF-I, we detected IGF-I expression in cerebellum of IGF-I Tg mice but not in mice lacking the IGF-I transgene (Figure 1C and 1D). Thus, by using the nestin/IGF-I mouse model, we accomplished our goal to target IGF-I overexpression to the neural precursors of the cerebellum, the potential medulloblastoma progenitor cells [17].

### Effects of IGF-I transgene expression on P5 cerebellum

**Figure 2:**
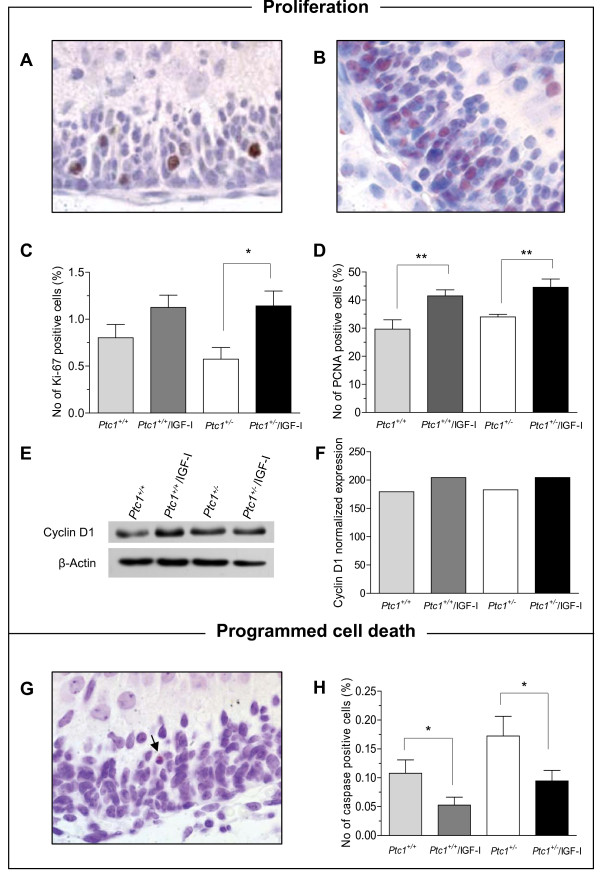
**Analysis of proliferation, programmed cell death and IGF-I signaling in P5 cerebellum of *Ptc1 *^*+/+*^, *Ptc1*^*+/-*^, *Ptc1*^*+/+*^/IGF-I Tg and *Ptc1*^*+/-*^/IGF-I Tg mice**. (A) Representative image of Ki-67 and (B) PCNA immunostaining. (C) Graphic representation of frequency of Ki-67, and (D) PCNA positive cells in the EGL. (E) Immunoblot analysis of expression of cyclin D1 with relative β-actin to control protein loading, and (F) relative graphic representation of densitometric analysis. (G) Representative image of caspase-3 positive cells in the EGL. (H) Graphic representation showing the frequency of caspase-3 positive cells in the EGL.

Next, we analyzed proliferation of GNPs in the setting of altered Shh and IGF signaling *in vivo*. These analyses were performed at postnatal day 5 (P5), when the IGF-I transgene is expressed at high level in the cerebellum [15]. We assessed the number of proliferating cells by immunohistochemistry using antibodies to the antigen encoded by the *Mki67 *gene (Ki-67) and the Proliferating Cell Nuclear Antigen (PCNA). The presence of the IGF-I transgene caused a significant increase in the number of Ki-67 positive GNPs in *Ptc1*^*+/- *^(1.14 *vs*. 0.57%; *P *= 0.0091) but not in *Ptc1*^*+/+*^mice (1.12 *vs*. 0.8%; *P *= 0.11; Figure 2A and 2C). Moreover, the presence of IGF-I transgene significantly increased the frequency of PCNA positive GNPs in both *Ptc1*^*+/+*^(41.6 *vs*. 29.7%; *P *= 0.0078) and *Ptc1*^*+/- *^mice (44.6 *vs*. 34.0%; *P *= 0.0055; Figure 2B and 2D). No significant differences in numbers of Ki-67 and PCNA positive cells were observed between *Ptc1*^*+/+*^and *Ptc1*^*+/- *^mice. By immunoblotting of isolated cerebellar extracts the expression of cyclin D1, a cell cycle regulatory protein was also increased, although not significantly, in the cerebellum of *Ptc1*^+/+ ^and *Ptc1*^*+/- *^mice carrying the IGF-I transgene (Figure 2E and 2F). Taken together, these data indicate that the IGF-I transgene strongly stimulates cell proliferation in the cerebellum.

During neural embryogenesis, about 50-70% of neural cells undergo programmed cell death leading to a massive loss of granule cells during active neurogenesis in the first three postnatal weeks of cerebellar development [18]. Several growth factors, including IGF-I, have been shown to modulate cell death in this population [19]. An inhibition of naturally occurring GNPs death may therefore represent a possible mechanism to sustain cell proliferation and tumor growth. We assessed the number of cells undergoing programmed cell death in the EGL by immunostaining using an antibody against cleaved caspase-3. The presence of the IGF-I transgene caused a significant reduction in the number of caspase-3 positive GNPs in *Ptc1*^*+/+*^/IGF-I Tg and *Ptc1*^*+/-*^/IGF-I Tg mice compared with the non transgenic mice (0.5% in *Ptc1*^*+/+*^/IGF-I Tg *vs*. 0.11% in *Ptc1*^*+/+*^; *P *< 0.005; 0.09% in *Ptc1*^*+/-*^/IGF-I Tg *vs*. 0.17% in *Ptc1*^*+/-*^; *P *< 0.005; Figure 2G and 2H). No significant difference was observed between *Ptc1*^+/+ ^and *Ptc1*^*+/- *^mice. Overall, our data indicate that the presence of IGF-I transgene increased proliferation rate and decreased programmed cell death in GNPs.

### Combined IGF-I transgene expression and Ptc1 mutation counteract differentiation of neural precursors

**Figure 3:**
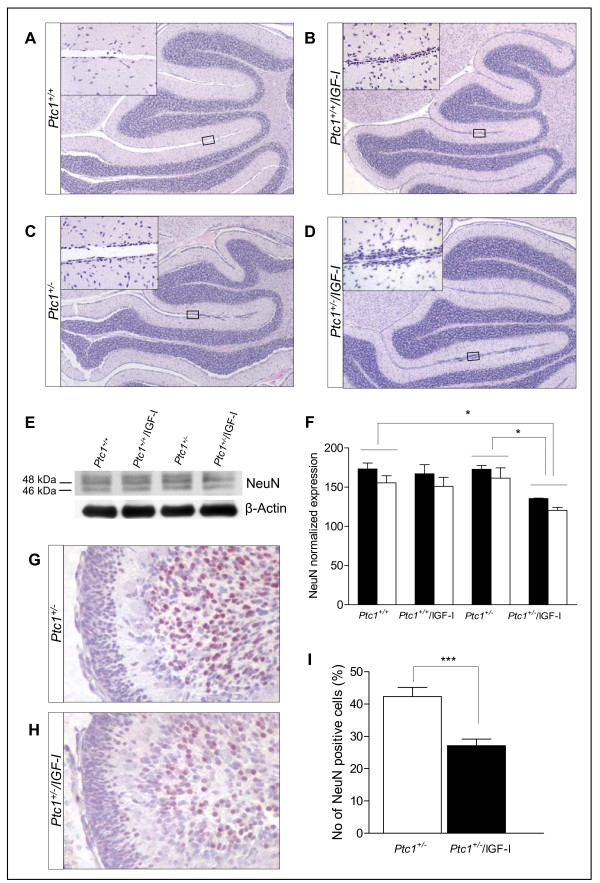
**Delayed differentiation in neural precursors caused by IGF-I altered expression**. (A) Morphologic analysis of H&E-stained sagittal sections of mouse cerebellum at P15, showing physiological absence of EGL in the cerebellum of *Ptc1*^+/+ ^mice. A thin 1-cell layer of EGL was present in the cerebellum of *Ptc1*^*+/+*^/IGF-I Tg (B), and *Ptc1*^*+/- *^mice (C). (D) A thicker 2-3-cells layer was observed in the EGL of *Ptc1*^*+/-*^/IGF-I Tg mice. (E) Western blot analysis showing the level of NeuN (48 and 46 kDa, solid and open square, respectively) expression in cerebellum from *Ptc1*^*+/+*^, *Ptc1*^*+/+*^/IGF-I Tg, *Ptc1*^*+/-*^, and *Ptc1*^*+/-*^/IGF-I Tg mice at P5, with relative β-actin to control protein loading. (F) Graphic representation of densitometric analysis. (G and H) Immunohistochemical analysis showing a decrease in the expression of NeuN in the IGL of the cerebellum of *Ptc1*^*+/-*^/IGF-I Tg mice (H) compared to *Ptc1*^*+/- *^mice (G). (I) Frequency of NeuN positive neurons in the IGL of *Ptc1*^*+/- *^and *Ptc1*^*+/-*^/IGF-I Tg mice. More than 5 × 10^3 ^granule neurons from 12 randomly selected digital images of the IGL (2 mice per genotype) have been examined.

Among the pleiotropic IGF-I activities during neurogenesis, IGF-I also affects neuronal differentiation, as well as possibly influencing neural stem cells. To study the effect of IGF-I transgene on GNPs differentiation we examined morphologic abnormalities in H&E-stained sagittal sections of the cerebellum at P15. At this age, in the cerebellum of wild-type mice GNPs have almost completed their migration into the IGL, and the EGL has ceased to exist (Figure 3A). Instead, a thin 1-cell layer of EGL was detected in the cerebellum of IGF-I Tg (Figure 3B) and *Ptc1*^*+/- *^mice (Figure 3C). A thicker EGL layer of 2-3-cells was observed in the cerebellum of double mutants (Figure 3D). These results indicate that Ptc1 mutation and transgenic IGF-I expression delayed differentiation of EGL progenitors in the cerebellum.

To evaluate the effect of IGF-I overexpression on neurogenesis, we examined the expression of NeuN, which marks postmitotic mature granule neurons in the IGL, by immunoblotting of isolated cerebellar extracts at P5. As shown in Figure 3E and 3F, the presence of IGF-I transgene in *Ptc1*^*+/- *^mice significantly decreased NeuN expression in the cerebellum of compound mutants (*P *< 0.05). In addition, immunostaining showed a strong decrease in expression of NeuN in *Ptc1*^*+/-*^/IGF-I Tg compared with *Ptc1*^*+/- *^mice (Figure 3G and 3H). Accordingly, quantization of NeuN positive neurons in the IGL showed a significant reduction in *Ptc1*^*+/-*^/IGF-I Tg (27%) compared with *Ptc1*^*+/- *^mice (42%; *P *= 0.0003; Figure 3I). This strongly suggests that IGF-I signaling cooperates with Shh deregulation in suppressing differentiation of granule progenitor cells from the active pool in the EGL. In this regard, a link between IGF signaling pathway and stem or progenitor cell potency has been recently highlighted by the finding that the number of cells expressing Sox9, a stem/progenitor cell biomarker, is decreased in intestinal crypts of IRS-1^-/-^/Min compared with IRS-1^*+/+*^/Min mice [20].

### Phenotype of nestin/IGF-I transgenic brains

**Figure 4:**
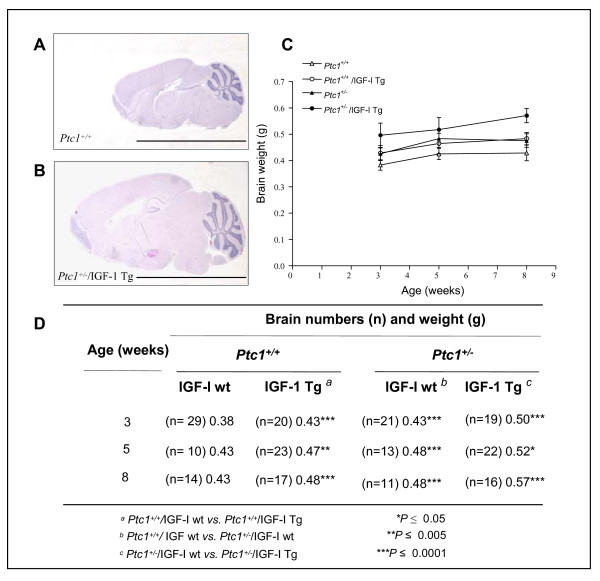
**Effect of IGF-I transgene on size and weight of mouse brain**. Representative H&E-stained sagittal sections of the brain from *Ptc1*^+/+ ^(A) and Ptc1^*+/-*^/IGF-I Tg mice (B) of 8 weeks of age, showing a large increase in brain size due to the combination of IGF-I transgene and *Ptc1 *heterozygous mutation. Bar = 5.0 mm. (C) Graphic representation of brain weights at 3, 5 and 8 weeks of age. (D) The presence of IGF-I transgene produced significant increase of brain weights both in *Ptc1*^+/+ ^and *Ptc1*^*+/- *^mice.

To evaluate the effect of the IGF-I transgene on brain growth, brains from *Ptc1*^*+/+*^, *Ptc1*^*+/+*^/IGF-I Tg, *Ptc1*^*+/-*^, and *Ptc1*^*+/-*^/IGF-I Tg mice of both sexes, at 3, 5 and 8 weeks of age were excised and weights determined. IGF-I acted to increase both size and weight of the developing brain. The largest difference in brain size was observed between *Ptc1*^+/+ ^and *Ptc1*^*+/-*^/IGF-I Tg mice at 8 weeks, as shown by representative H&E-stained sagittal sections (Figure 4A and 4B). Importantly, the cerebellum was among the brain regions showing a more marked overgrowth. Compared with *Ptc1*^+/+ ^littermates, the presence of IGF-I transgene produced significant brain weight increases at all time points examined (9.3-13.2%; *P *≤ 0.005; Figure 4C and 4D). In comparison with *Ptc1*^+/+ ^littermates, significant increments were also produced by heterozygosity of the *Ptc1*^*+/- *^gene (11.6-13.2%; *P *≤ 0.05). In addition, the presence of IGF-I transgene in *Ptc1*^*+/- *^mice caused a further significant increase in brain weight compared with *Ptc1*^*+/- *^littermates (8.3-18.8%; *P *≤ 0.05; Figure 4C and 4D). Despite the striking effect of the IGF-I transgene on brain size, the overall neural development was relatively normal and transgenic mice showed normal appearance and behavior. On the whole, these results indicate that the presence of either the IGF-I transgene or of the *Ptc1 *mutation leads to macrocephaly, as brains were larger and weighted significantly more compared with littermate controls. Furthermore, the increment in brain weight observed in *Ptc1*^*+/- *^mice carrying the IGF-I transgene suggests an independent and cooperative effect of Shh and IGF-I pathways in brain development.

### IGF-I has tumor promotion activity in medulloblastoma tumorigenesis

**Figure 5:**
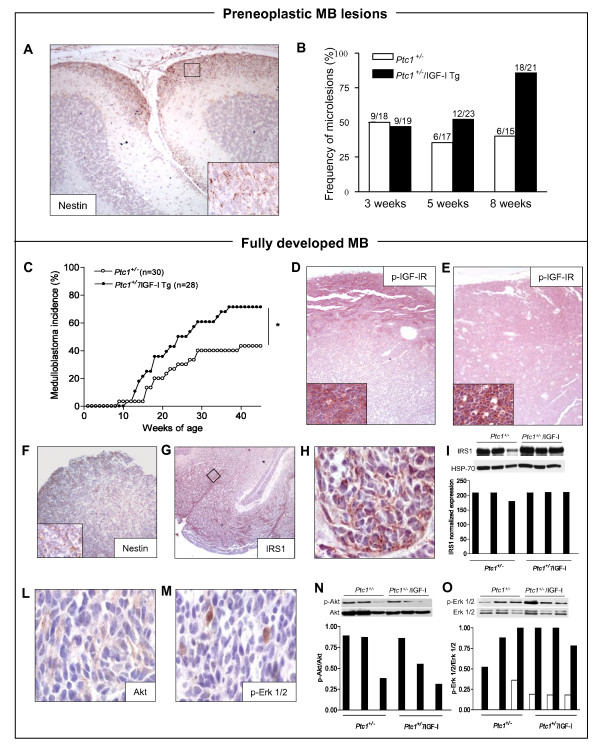
**Acceleration of tumor development in the cerebellum of *Ptc1*^*+/- *^mice expressing IGF-I transgene under control of the nestin promoter**. (A) Representative immunohistochemical analysis of nestin in preneoplastic lesions detected in *Ptc1*^*+/- *^cerebellum at 3 weeks of age. (B) Frequency of preneoplastic medulloblastoma lesions in cerebellum of *Ptc1*^*+/- *^and *Ptc1*^*+/-*^/IGF-I Tg mice of 3, 5 and 8 weeks of age. (C) The IGF-I transgene caused a significant enhancement in medulloblastoma development (71% in *Ptc1*^*+/-*^/IGF-I Tg mice *vs*. 43% in *Ptc1*^*+/- *^mice; *P *< 0.05). (D and E) Representative immunostaining for phosphotyrosine 1316 (*pY *1316) IGF-IR in medulloblastomas from *Ptc1*^*+/-*^mice and *Ptc1*^*+/-*^/IGF-I Tg mice. Insets in (D) and (E), higher magnification (100×). (F) Analysis of nestin expression in medulloblastomas from *Ptc1*^*+/- *^mice. (G) Representative immunostaining for IRS1 in medulloblastomas from *Ptc1*^*+/- *^mice. (H) Higher magnification (100×). (I) Immunoblotting of IRS1 in medulloblastomas from *Ptc1*^*+/- *^and *Ptc1*^*+/-*^/IGF-I Tg mice, with relative HSP-70 to control protein loading, and graphic representation of densitometric analysis (lower panel). (L and M) Representative immunostaining for Akt and p-Erk1/2 in medulloblastomas from *Ptc1*^*+/- *^mice. Evaluation of Akt/Pkb (N) and Erk1/2 (O) phosphorylation status by immunoblotting in medulloblastomas from *Ptc1*^*+/- *^and *Ptc1*^*+/-*^/IGF-I Tg mice, and relative graphic representation of densitometric analysis.

As a next step, we determined the frequency of early and fully developed medulloblastoma stages in the cerebellum of the F1 progeny of crosses between *Ptc1*^*+/- *^and IGF-I Tg mice. During postnatal cerebellar development, differentiating GNPs complete their migration from the EGL to IGL by the third postnatal week. The presence of EGL remnants in the cerebellum of *Ptc1*^*+/- *^mice, which persist after the 3rd week of age, is considered indicative of a differentiation defect of GNPs, suggestive of a preneoplastic condition [8,9,12]. Notably, ectopic EGL areas from 3-week old *Ptc1*^*+/- *^mice markedly expressed nestin, thus assuring the expression of the nestin/IGF-I transgene from the initial steps of the tumorigenic process (Figure 5A). To determine whether IGF-I affects early tumor development, brains from asymptomatic *Ptc1*^*+/+*^, *Ptc1*^*+/+*^/IGF-I Tg, *Ptc1*^*+/- *^and *Ptc1*^*+/-*^/IGF-I Tg mice at 3, 5 or 8 weeks were histologically examined and cerebellar pathology was assessed (Figure 5B). At 3 weeks of age, medulloblastoma precursor lesions were evident in 50% (9/19) of *Ptc1*^*+/-*^/IGF-I Tg and (9/18) *Ptc1*^*+/-*^mice. At 5 weeks, 52.2% (12/23) of *Ptc1*^*+/-*^/IGF-I Tg mice presented cerebellar abnormalities compared with 35.3% (6/17) of *Ptc1*^*+/- *^mice. The largest effect of IGF-I transgene was evident at 8 weeks, when a significant increase of preneoplastic lesions was observed in *Ptc1*^*+/-*^/IGF-I Tg compared with *Ptc1*^*+/- *^mice (85.7%, 18/21, *vs*. 40%, 6/15; *P *= 0.01). No ectopic EGL areas were observed in the cerebellum of *Ptc1*^+/+ ^and *Ptc1*^*+/+*^/IGF-I Tg mice. These findings suggest that the IGF-I transgene, by protracting the susceptible phase of the cerebellum to development of preneoplastic areas in the cerebellum of *Ptc1*^*+/- *^mice, fosters *de novo *formation of EGL lesions.

The observation that IGF-I promotes the initial steps of medulloblastoma growth in *Ptc1*^*+/- *^mice prompted us to examine its influence on development of advanced tumors. To this aim, the F1 progeny of crosses between *Ptc1*^*+/- *^mice and IGF-I Tg mice was placed on a lifetime study and brain tumor development was monitored. Notably, the IGF-I transgene produced a significant acceleration of medulloblastoma development (Figure 5C). By 15 weeks, 7 of 28 (25.0%) *Ptc1*^*+/-*^/IGF-I Tg mice had developed medulloblastoma compared with 1 of 30 (3.3%) *Ptc1*^*+/- *^mice. At the end of the experiment, 20 of 28 (71.4%) *Ptc1*^*+/-*^/IGF-I Tg mice developed medulloblastomas compared with 13/30 (43.3%,) *Ptc1*^*+/- *^mice (P < 0.05). These data highlight the influence of the IGF-I transgene on the malignant potential of preneoplastic EGL. Up to 50% of young (3-8 wks) *Ptc1*^*+/- *^mice show presence of precursor lesions in cerebellum, and given a final medulloblastoma incidence of 43%, about 86% of these preneoplastic areas have potential to give rise to full tumors. The IGF-I transgene caused a significant 2-fold increase in the frequency of preneoplastic lesions (86%) in *Ptc1*^*+/-*^/IGF-I Tg mice. Based on a final medulloblastoma incidence of 71%, the conversion rate of preneoplastic lesions was unaltered compared to *Ptc1*^*+/- *^mice (83% *vs*. 86%). This observation suggests that the IGF-I transgene results in *de novo *formation of preneoplastic lesions but does not modify their rate of progression to full tumors. However, IGF-I *per se *does not exert a tumor initiating activity *in vivo*, as no medulloblastomas developed in *Ptc1*^*+/+*^/IGF-I Tg mice.

Histology of medulloblastomas revealed no major morphological differences with respect to the presence of IGF-I transgene (data not shown). Immunohistochemistry of tumors from *Ptc1*^*+/- *^mice showed a strong expression of p-IGF-IR - the active IGF-IR form - localized on the outer part of the tumors, suggesting that IGF signaling is required for medulloblastoma growth in the *Ptc1*^*+/- *^mouse model (Figure 5D). Interestingly, tumors from *Ptc1*^*+/-*^/IGF-I Tg mice revealed a strong and uniform p-IGF-IR staining throughout the tumor mass (Figure 5E). This probably reflects a generalized expression of the nestin/IGF-I transgene that follows the spatial expression pattern of the *nestin *native gene throughout the tumor (Figure 5F). We also examined by immunohistochemistry the expression of IRS1, Akt/Pkb and Erk1/2 kinases, downstream mediators of the IGF-I signaling pathway, in medulloblastoma samples from *Ptc1*^*+/- *^and *Ptc1*^*+/-*^/IGF-I Tg mice (n = 3). All the tumors from single and compound mutants showed IRS1, Akt and Erk 1/2 expression (Figure 5G, H, L and 5N), indicating that IGF signaling is required to maintain tumor growth *in vivo*. By immunoblotting, we determined IRS1 expression, as well as total and phosphorylated Akt/Pkb and Erk 1/2 protein levels. All the tumors strongly expressed IRS1 irrespective of the presence of IGF transgene, and showed a large intertumor variability in the activation of Akt/Pkb and Erk 1/2 protein that did not correlate with transgenic IGF-I expression (Figure 5I, N and 5O). To further investigate whether the IGF-I transgene influences the mechanisms of tumorigenesis, we assayed loss of the wild-type *Ptc1 *allele, a prerequisite for the biological switch to malignancy of early cerebellar lesions in *Ptc1*^*+/- *^mice [9], in medulloblastoma from compound mutants. Sequence analysis of tumor DNA showed that, similar to medulloblastomas from *Ptc1*^*+/- *^mice, tumors from *Ptc1*^*+/-*^/IGF-I Tg mice (n = 3) also showed lack of wild type *Ptc1 *(data not shown). Altogether, these data indicate that IGF-I strongly modulates the penetrance of medulloblastomas but not the molecular pathogenesis of tumors in the *Ptc1*^*+/- *^mouse model.

## Discussion

Normal proliferation of GNPs in the cerebellum is dependent upon Shh and IGF-I signaling, and deregulation of both pathways is implicated in medulloblastoma [4,5,21-23]. Constitutive activation of the Shh pathway - frequently due to inactivating mutations of *Ptc1 *- has been shown in approximately 30% of human medulloblastomas [1]. Molecular oncology studies in humans and mice strongly implicated IGFs as additional causative factors for medulloblastoma. In fact, increased expression levels of IGF-II have been shown in human medulloblastomas, and overexpression of IGF-IR and IGF-I mRNA was observed in medulloblastoma cell lines [24,25]. In addition, a strong synergy between IGF and Shh signaling pathways has been demonstrated by using the RCAS/*tv-a *system, in which combined expression of IGF-II and Shh was shown to induce medulloblastoma at a significantly higher incidence compared with Shh alone [23]. In this system, however, gene transfer is performed in the cerebella of newborn mice, thus hampering investigations on the early effects of such a synergy on neural precursors, the proposed cells of origin of medulloblastoma. In the present study, we cross-bred nestin/IGF-I Tg mice, in which transgene expression starts prenatally and is detectable in the cerebellar primordium as early as embryonic day 13 [15], with *Ptc1*^*+/- *^mice, a faithful model of medulloblastoma recapitulating the histopathology of the human tumor. Importantly, the use of this novel genetic cross offers the opportunity to study how interactions between Shh and IGF-I signaling, starting during embryonic life, affect development and neoplastic growth of neural precursors in neonatal cerebellum.

As already reported in a different line of IGF-I Tg mice [19], we show here that transgenic expression of IGF-I in the cerebellum during development produced a hyperplastic EGL, characterized by neural precursors exhibiting increased proliferation and decreased programmed cell death. We also report a novel effect of the IGF-I transgene in our system, *i.e*., a marked differentiation defect, as shown by a reduced expression of NeuN and a delayed disappearance of neural progenitors from the EGL pool. Furthermore, in line with a previous report that IGF-I promotes brain overgrowth by stimulating neural cell proliferation and inhibiting apoptosis in the cerebral cortex [15], we found that nestin/IGF-I Tg mice exhibited a marked generalized brain overgrowth that also includes the cerebellum. Moreover, we provide evidence that IGF-I overexpression in cerebellum cooperates with deregulation of the Shh pathway to further enhance brain overgrowth in double mutants, and to accelerate medulloblastoma development by significantly increasing the incidence of early, as well as full medulloblastoma stages in *Ptc1*^*+/-*^/IGF-I Tg compared with *Ptc1*^*+/- *^mice. These findings identify a novel synergy of IGF-I and Shh signaling pathways during cerebellum development and confirm, in this new genetic cross, the robust cooperation between IGF-I and Shh signaling in medulloblastoma tumorigenesis.

Our findings also suggest that brain overgrowth and increased tumor formation may stem from a common mechanism favoring survival and proliferation of neural precursors over terminal differentiation, thus stressing the link between aberrant activation of developmental pathways and tumorigenesis. On the other hand, we have previously shown that the overexpression of PC3, a gene that acts as a switch from proliferative to neuron-generating cell fate, causing a marked increase of differentiation in neuronal precursors and impairment of cerebellar development [26], significantly inhibited medulloblastoma tumorigenesis in *Ptc1*^*+/- *^mice [27]. Taken together these findings indicate that sets of genes controlling cell growth and differentiation may coordinately modulate developmental patterns and susceptibility to cancer in CNS. In this respect, it is also worth noting that height and weight at birth, relating to IGF-I concentration in umbilical cord [28], have been found to positively correlate with increased cancer risk in humans [29-32].

Medulloblastomas from *Ptc1*^*+/- *^and *Ptc1*^*+/-*^/IGF-I Tg mice both express active IGF-IR, although with a different staining pattern probably reflecting the generalized expression of the nestin/IGF-I transgene in tumors from double mutants. Medulloblastomas from *Ptc1*^*+/- *^and *Ptc1*^*+/-*^*/*IGF-I Tg mice also express IRS1, and show Akt and Erk 1/2 activation, demonstrating a functional role for the IGF-I signaling system in medulloblastoma formation. Furthermore, IGF-I transgenic expression does not influence the morphological characteristics of the tumors, nor the genetic events in tumorigenesis, as *Ptc1 *inactivation represents the critical event in medulloblastoma development in both *Ptc1*^*+/- *^and *Ptc1*^*+/-*^/IGF-I Tg mice. Altogether, these results indicate that IGF-I modulates tumor development in CNS of *Ptc1*^*+/- *^mice but does not alter the pathogenesis of tumor development.

A key question relative to the mechanism of cancer promotion by IGF-I is whether it involves (i) tumor initiation, through a pro-survival effect, leading to survival of a mutated cell, or (ii) malignant conversion, through a mitogenic effect that facilitates progression of precancerous stages [33]. In this respect, our novel mouse cross has proven useful. Through analysis of preneoplastic cerebellar lesions we show that, although IGF-I overexpression is not by itself carcinogenic in CNS, it can nevertheless increase tumor penetrance in a genetically susceptible model of human medulloblastoma by increasing the number of mice bearing medulloblastoma precursor lesions. On the contrary, we show that the rate of conversion of early to fully malignant tumor stages is not modified by IGF-I. Collectively, these findings suggest that IGF-I may have a role as a risk factor in susceptible individuals. Therefore, IGF-I levels should be regarded as a tumor modifying factor concurring to determine individual susceptibility to cancer. From a more general standpoint, if such a basic science findings translate to the human population, they might have important general implications for tumorigenesis. In fact, IGF-I signaling is also relevant to neoplasia in a number of other tissues such as peripheral nervous system, skin, and prostate [34-38], and epidemiological studies have linked high circulating levels of IGF-I with increased cancer risk in breast, prostate and colon cancer [39-44]. Interestingly, the hypothesis of IGF-I as modifier of disease risk is supported by a recent report showing a strong association of IGF1 CA repeat polymorphism and early onset of colorectal cancer in hereditary non-polyposis colorectal cancer patients [45]. These observations provide a solid foothold to pursue this topic further.

## Conclusions

In summary, we made use of a novel genetic mouse cross of deregulated Shh and IGF-I signaling to show that brain growth patterns and tumor growth are modulated by IGF-I host physiology. We have also identified increased survival and proliferation and suppression of differentiation in neural precursors as the underlying biological mechanisms linking IGF-I signaling with brain overgrowth and tumor development in a powerful mouse model of medulloblastoma. Finally, we have shown an important role of IGF-I altered expression in the initiation and maintenance of early lesions *en route *to medulloblastoma.

Understanding the molecular events responsible for the normal developmental process of neural progenitor cells, and how these are altered to sustain the tumorigenic process is a necessary first step towards identification of potential targets for therapeutic intervention.

## Methods

### Animals and genotyping

Mice lacking one *Ptc1 *allele (*Ptc1*^*neo6-7/+*^, named *Ptc1*^*+/- *^throughout the text) generated through disruption of exons 6 and 7 in 129/Sv embryonic stem cells [46] and maintained on C57BL/6 background were crossed to IGF-I transgenic mice maintained on the same background and overexpressing *Homo Sapiens *IGF-I (A.J. D'Ercole, University of North Carolina at Chapel Hill). The mouse lines and F1 progeny resulting from crossings were genotyped using primers specific for the *neo *insert and *wt *regions of the *Ptc1 *gene as described [46], and primers specific for the human IGF-I transgene: 5'-GGA CCG GAG ACG CTC TGC GG -3' and 5' - CTG CGG TGG CAT GTC ACT CT - 3'.

Animals were housed under conventional conditions with food and water available *ad libitum *and a 12-h light cycle. Experimental protocols were reviewed by the Institutional Animal Care and Use Committee.

### RNA extraction and reverse transcription-polymerase chain reaction

Total RNA was isolated from cerebella at postnatal day 5 (P5) using SV Total RNA Isolation System (Promega, Madison, WI) and stored at -80°C until further processing. Total RNA (2 μg) was reverse transcribed using RETROscript (Ambion, Inc., Austin, TX) according to the instructions of the manufacturer. The primer pairs used were sense 5'- TGG ATG CTC TTC AGT TCG TG - 3' and antisense 5'- CCT GCA CTC CCT CTA CTT GC -3' corresponding to the *Homo Sapiens *IGF-I transgene cDNA, yielding a 265-bp product.

### Histological analysis and tumor quantification

Mice were observed daily for their whole lifespan. Upon decline of health (i.e., severe weight loss, paralysis, ruffling of fur, or inactivity), they were euthanized and autopsied. Brains were fixed in 4% buffered formalin. Samples were then embedded in paraffin wax according to standard techniques, sectioned and stained with H&E. Medulloblastoma incidence was expressed as the percentage of mice with the tumor.

### Tissue collection

Asymptomatic *Ptc1*^*+/+*^, *Ptc1*^*+/+*^/IGF-I Tg, *Ptc1*^*+/- *^and *Ptc1*^*+/-*^/IGF-I Tg mice were euthanized at P5 or P15 and brains were fixed in 4% buffered formalin and/or preserved at -80°C. For determination of preneoplastic stages, asymptomatic mice were also euthanized at 3, 5 or 8 weeks. The brains were removed, weighted and fixed in 4% buffered formalin to evaluate the incidence of hyperplastic areas in the cerebellum. In all, 18 sections were examined for each cerebellum with an interval of 70 μm.

### Immunohistochemistry and immunoblotting analysis

Immunohistochemistry analysis was carried out on 4-μm thick paraffin sections of cerebellum at P5 or on sections of medulloblastoma samples. Antibody-antigen complexes were visualized using a horseradish peroxidase-conjugated secondary antibody and the DAB chromogen system (Dako North America, Inc, Carpinteria, Ca). Immunohistochemistry analysis of monoclonal antibody against NeuN (Millipore Billerica, MA) and PCNA (Ab-1/PC-10, Calbiochem, Germany) was performed using the HistoMouse MAX Kit (Zymed Laboratories, San Francisco, CA) according to the manufacturer's instructions.

For immunoblotting, proteins (30 μg) were extracted from a pool of 2 cerebella (P5) per genotype, and from medulloblastomas developed in *Ptc1*^*+/- *^and *Ptc1*^*+/-*^/IGF-I Tg mice [47]. Proteins were visualized with horseradish peroxidase-conjugated secondary antibodies (Santa Cruz Biotechnology, Santa Cruz, CA) followed by chemiluminescence detection (SuperSignal West Pico Chemiluminescent Substrate; Pierce, Rockford, IL). Protein levels were quantified by densitometric analysis using Scion Image Beta 4.02 software package (Scion Corporation, Frederick, MD). We used mouse anti-β-actin or anti-Heat Shock Protein 70 (HSP-70) antibody (Sigma-Aldrich Inc., St Louis, MO) to control protein loading. Two to three blots were run for each set of samples.

Other antibodies used include rabbit polyclonal antibody against nestin (Abcam Ltd, Cambridge, UK), Ki-67 (Novocastra, Novocastra Laboratory, Newcastle, UK), cleaved caspase-3 (Asp175), IGF-I receptor β, p-IGF-I receptor β, Erk1/2, p-Erk1/2, p-Akt (Ser473), total Akt, all from Cell Signaling (Beverly, MA), IRS-1 (Santa Cruz Biotechnology), goat polyclonal antibody against human IGF-I antibody (R&D System, MN), monoclonal antibody against cyclin D1 (Santa Cruz Biotechnology).

### Analyses of proliferation and programmed cell death

Paraffin sections of cerebellar tissue of pups at P5 were cut at 4 μm thickness. Immunohistochemical analysis of Ki-67, PCNA and caspase-3 were performed on brain samples. Digital images of the entire midsagittal cerebellar section from 3 mice were collected by IAS image-processing software (Delta Sistemi, Rome, Italy). Ki-67-, PCNA- and caspase-3 positive cells in the EGL were counted. Rates of proliferation and apoptosis were calculated as the percentage of positively stained cells relative to the total number of cells of the EGL.

### LOH analysis at the Ptc1 locus

DNA was extracted from tumors and normal tissue of *Ptc1*^*+/- *^(n = 3) and *Ptc1*^*+/-*^/IGF-I Tg mice (n = 3) using Wizard SV Genomic DNA Purification System (Promega). LOH analysis was performed as described [47].

### Statistics

Statistical comparisons were made using Student's t-test and Fisher exact test. *P *values < 0.05 were considered statistically significant.

## Competing interests

The authors declare that they have no competing interests.

## Authors' contributions

All authors participated in the design of the study. MS, MT, EP and SL performed the experimental work. MM, VDM, SR and AS contributed to data analysis and interpretation. SP conceived the study and wrote the manuscript. All the authors read, revised and approved the final manuscript.
